# Supplementary material for: A Critical View of the Application of the APEX Software (Aqueous Photochemistry of Environmentally-Occurring Xenobiotics) to Predict Photoreaction Kinetics in Surface Freshwaters
Source: Molecules. 2019 Dec 18;25(1):9. doi: 10.3390/molecules25010009 (PMC7017383; doi:10.3390/molecules25010009)
Supplement: Supplementary file 1 [file molecules-25-00009-s001.zip › 3.2.4_gcc-4.4.0.zip!/3.2.4_gcc-4.4.0/3.2.4_gcc-4.4.0/doc/octave/html/interpreter/Paging-Screen-Output.html]

Paging Screen Output - Untitled


Up: Terminal Output


---

##### 14.1.1.1 Paging Screen Output

When running interactively, Octave normally sends any output intended
for your terminal that is more than one screen long to a paging program,
such as `less` or `more`. This avoids the problem of having a
large volume of output stream by before you can read it. With
`less` (and some versions of `more`) you can also scan forward
and backward, and search for specific items.

Normally, no output is displayed by the pager until just before Octave
is ready to print the top level prompt, or read from the standard input
(for example, by using the `fscanf` or `scanf` functions).
This means that there may be some delay before any output appears on
your screen if you have asked Octave to perform a significant amount of
work with a single command statement. The function `fflush` may be
used to force output to be sent to the pager (or any other stream)
immediately.

You can select the program to run as the pager using the `PAGER`
function, and you can turn paging off by using the function
`more`.

— Command: **more**  
— Command: **more** on  
— Command: **more** off  
> Turn output pagination on or off. Without an argument, `more`
> toggles the current state.
> The current state can be determined via `page_screen_output`.

— Built-in Function: val = **PAGER** ()  
— Built-in Function: old\_val = **PAGER** (new\_val)  
> Query or set the internal variable that specifies the program to use
> to display terminal output on your system. The default value is
> normally `"less"`, `"more"`, or
> `"pg"`, depending on what programs are installed on your system.
> See Installation.
>
>
>
> **See also:** more, page\_screen\_output, page\_output\_immediately, PAGER\_FLAGS.

— Built-in Function: val = **PAGER\_FLAGS** ()  
— Built-in Function: old\_val = **PAGER\_FLAGS** (new\_val)  
> Query or set the internal variable that specifies the options to pass
> to the pager.
>
>
>
> **See also:** PAGER.

— Built-in Function: val = **page\_screen\_output** ()  
— Built-in Function: old\_val = **page\_screen\_output** (new\_val)  
> Query or set the internal variable that controls whether output intended
> for the terminal window that is longer than one page is sent through a
> pager. This allows you to view one screenful at a time. Some pagers
> (such as `less`—see Installation) are also capable of moving
> backward on the output.

— Built-in Function: val = **page\_output\_immediately** ()  
— Built-in Function: val = **page\_output\_immediately** (new\_val)  
> Query or set the internal variable that controls whether Octave sends
> output to the pager as soon as it is available. Otherwise, Octave
> buffers its output and waits until just before the prompt is printed to
> flush it to the pager.

— Built-in Function: **fflush** (fid)  
> Flush output to fid. This is useful for ensuring that all
> pending output makes it to the screen before some other event occurs.
> For example, it is always a good idea to flush the standard output
> stream before calling `input`.
>
> `fflush` returns 0 on success and an OS dependent error value
> (−1 on unix) on error.
>
>
>
> **See also:** fopen, fclose.
